# Soft X-ray beamline BL1N2 at Aichi Synchrotron Radiation Center and its industrial use

**DOI:** 10.1107/S1600577523004423

**Published:** 2023-06-20

**Authors:** Harue Sugiyama, Haruki Murase, Toyokazu Nomoto, Yoshikazu Takeda

**Affiliations:** a Aichi Synchrotron Radiation Center, Aichi Science and Technology Foundation, 250-3 Minamiyamaguchi-cho, Seto, Aichi 489-0965, Japan; University of Tokyo, Japan

**Keywords:** soft X-ray XAFS, mirror contamination, industrial use

## Abstract

BL1N2 is a soft X-ray XAFS (X-ray absorption fine structure) beamline that is well suited for industrial use. User service started in 2015. The beamline is a grazing optical system with a pre-mirror, an inlet slit, two mirrors for three gratings, an outlet slit and a post-mirror. Light of 150 eV to 2000 eV is available, and *K*-edge measurements of elements from B to Si are covered. The O *K*-edge is most often measured; transition metals such as Ni and Cu at their *L*-edges and lanthanoids at their *M*-edges are also often measured. Here, basic information about BL1N2, the effect of ageing by synchrotron radiation to remove mirror contamination, and a compatible sample handling system and transfer vessels to allow a one-stop service at three soft X-ray beamlines at AichiSR are described.

## Abstract 2

Basic information about beamline BL1N2, the effect of ageing by synchrotron radiation to suppress mirror contamination, and efforts to support industrial use are reported.

## 1. Introduction

Aichi Synchrotron Radiation Center (AichiSR) was built in Aichi, Japan, in 2012 and started user service in March 2013 with six beamlines (Takashima *et al.*, 2016 ▸; Kunieda *et al.*, 2022 ▸). The light sources are four superconducting bending magnets (superbends, 5 T), eight normal-conducting bending magnets (normal-bends, 1.4 T), and one undulator in the 1.2 GeV top-up operation storage ring. The first six beamlines are: a hard X-ray XAFS (X-ray absorption fine structure) beamline, BL5S1; a tender X-ray XAFS beamline, BL6N1; a VUV spectroscopy beamline, BL7U; a powder X-ray diffraction beamline, BL5S2; a surface diffraction beamline, BL8S1; and a small-angle X-ray scattering beamline, BL8S3.

Three beamlines, BL7U, BL6N1 and BL5S1, were originally designed to cover the *K*-edge absorption energies of elements from Li (55 eV) to Mo (20 keV) continuously. BL6N1 was expected to cover the energy range from 600 eV to 4000 eV with a double-crystal monochromator using crystals Si, Ge, InSb, YB66, β-alumina, KTP and/or beryl, similar to beamline BL2A at UVSOR (Hiraya *et al.*, 1992 ▸). However, during the commissioning period of this beamline, it was found that the diffraction efficiency of YB66 was very low and that β-alumina was severely damaged at the footprint (a ‘white’ crescent was observed) and no X-rays were detected at the end-station. At this moment we gave up trying the other crystals, KTP and beryl. This meant that the energy range 1000–1800 eV covering the Na, Mg and Al *K*-edges was missing in these beamline setups, though Al and Mg are essential for weight reduction of industrial products – this is currently a critical issue for a low-carbon society. Therefore, we designed a beamline, BL1N2, that covers, at least, the missing energy range using a diffraction grating. Fortunately, the construction proposal for this beamline was accepted as a member of the Photon-Beam Platform Project (we joined the Project from the fiscal year 2021 and our construction proposal for BL1N2 was included in the supplementary budget for the Platform Project), supported by the Japanese Ministry of Education, Culture, Sports, Science and Technology. The beamline consists of three grating mirrors: G1 and G3 with 500 lines mm^−1^, and G2 with 1000 lines mm^−1^. In total, this beamline covers the whole energy range from 150 eV to 2000 eV. Therefore, XAFS measurements are possible at the *K*-edges of elements with atomic numbers 5 to 14 such as B, C, O, F, Ne, Na, Mg, Al and Si, at the *L*-edges of elements with atomic numbers 16 to 36 that include important metals such as Ti, Cr, Mn, Fe, Co and Ni, and at the *M*-edges of elements with atomic numbers 37 to 76 that include most of the lanthanoids.

In the 2021 fiscal year, the most measured element at BL1N2 was oxygen. Oxygen is often measured not only in organic materials but also with light metals such as Al and Ti, transition metals such as Fe and Ni, and lanthanoids. Lanthanoids are a crucial elemental group for actively developing battery materials and LED materials in the context of realizing a low-carbon society. Lanthanoids are in a group of elements whose *M*-edge spectra better manifest chemical properties such as valence changes than *L*-edge spectra. Since the analytical energy band of Al and lanthanoids falls into the valley of two monochromatic technologies, between a diffraction grating monochromator and a crystal monochromator, examination of these features is not possible at most of the existing beamlines in Japan. BL1N2, equipped with grazing-incidence optics to fill the gap, is well recognized and often selected by users.

The measurement elements at BL1N2 are diverse. Many users of BL1N2 measure multiple elements during a single use. In 2021, the materials measured were 21% organic chemical materials, 21% batteries or catalyst materials, and 13% metallic materials. Universities and other public institutes used 30% of beam time and 70% was used by industries. This ratio has remained about the same over recent years.

## 2. Basic information of BL1N2

**Figure 1:**
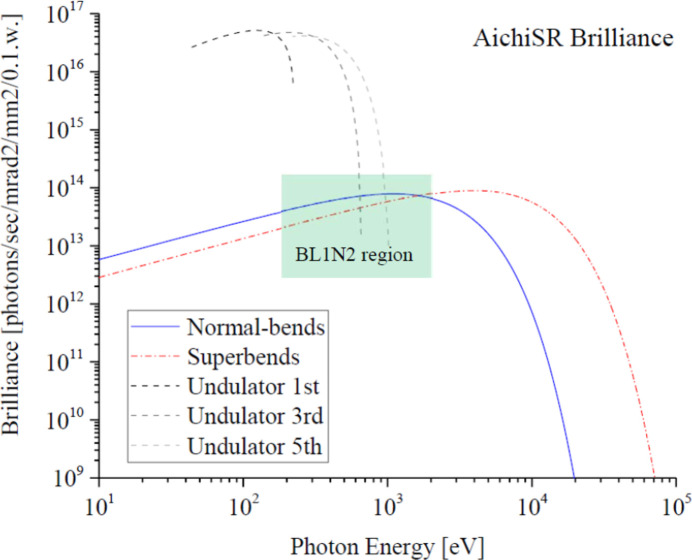
Brilliance curves from the normal-conducting bending magnets (normal-bends, solid line), from the superconducting bending magnets (superbends, dash-dotted line) and from the undulator (dashed line) of AichiSR.

The brilliance curves of the normal-bends (solid line) and the superbends (dash-dotted line) are shown in Fig. 1 ▸. At BL1N2, for which the light source is the normal-bending magnet with the magnetic field 1.4 T, the normal-bends cover the energy range from 150 eV to 2000 eV around the highest portion of the blue brilliance curve.

### 2.1. Optical components

**Figure 2:**
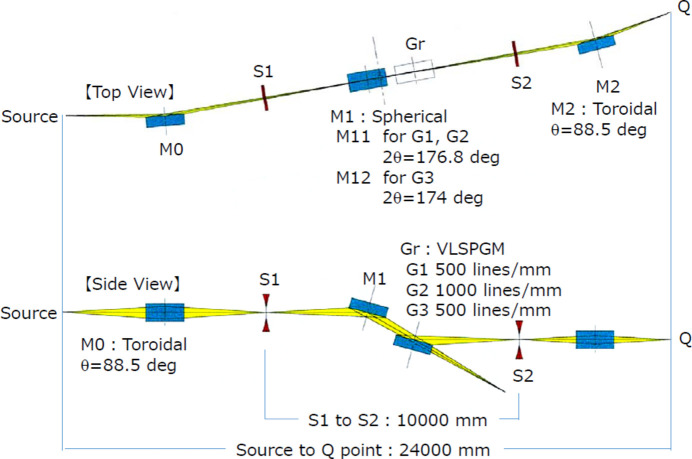
Outline of the optical system of BL1N2. Top view (top) and side view (bottom).

The optics of BL1N2 consists of a toroidal pre-mirror (M0), an inlet slit (S1), two spherical mirrors for gratings (M11 and M12), three VLS-PGM type plane gratings (G1, G2 and G3), an outlet slit (S2) and a toroidal post-mirror (M2). Fig. 2 ▸ shows the configuration of the BL1N2 optics, and Tables 1 ▸ and 2 ▸ show the specifications of these mirrors. Table 3 ▸ shows the focal lengths. The total length is 24000 mm from the light source point to the final focus (Q point). The entire optical system has a 1:1 focal system.

### 2.2. Grazing incidence and choice of coating metals

There are two notable features of the optical system. Firstly, despite the fact that M0 is a toroidal mirror, the incident angle is as large as 88.5° (grazing angle of 1.5°). In a toroidal mirror, a single mirror focuses in two directions – sagittal and tangential. Precise alignment is required to pass light through a grazing toroidal mirror. Still, this method was chosen because it requires sufficient flux up to 2000 eV to provide the Al *K*-edge XAFS measurements.

The reflectance of Au, which is the coating material of the mirror, is about 50% at 2000 eV with an incident angle of 88.4°. The amount of light that can pass through two 50% reflective mirrors is 50% × 50%, which is 25%. As the number of mirrors increases, the amount of light decreases. For these two reasons, at BL1N2, M0 had to cover the sagittal and tangential directions with one mirror to suppress the attenuation of the flux, while the incident angle had to be larger than 88.4°. For the same reason, M11 also adopts an included angle of 176.8° (incident angle 88.4°, grazing angle 1.6°).

**Figure 3:**
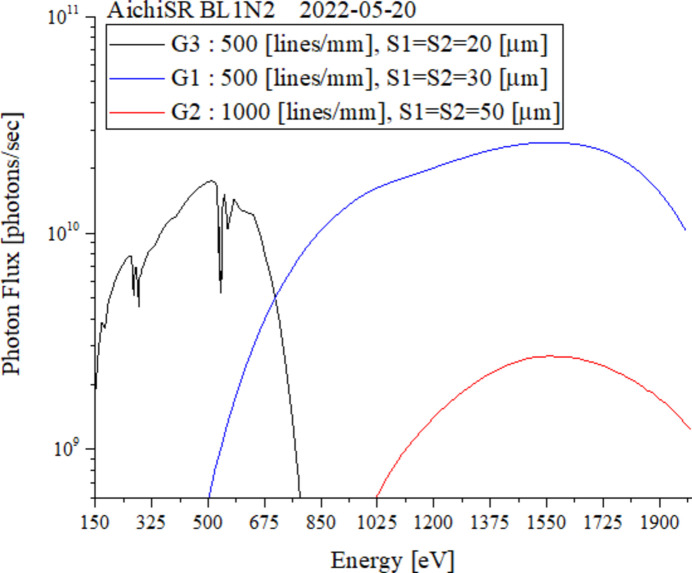
Photon flux of BL1N2 using G1, G2 and G3 setups. G2 is used when a high energy resolution over 1000 eV is required. The Mn *L*-edge (approximately 640 eV) uses G3 and G1 at the boundary line.

Secondly, the mirror used in the setup of G3 is Ni-coated to cover the lower energy from 150 eV to 700 eV. The reflectance of Ni is more than that of Au in this energy region. Moreover, light with higher energy than the Ni *L*-edge at 850 eV is attenuated by orders of magnitude. Therefore, it is possible to provide carbon and oxygen XAFS (∼270 eV to 600 eV) without difficulty while using a light source with a maximum brilliance at 1000 eV. In particular, when measuring oxygen, the sample is often a compound of oxygen and sodium or a lanthanide. Therefore, it is important to be able to cut off high-order light around 1000 eV. As a result, the flux observed at BL1N2 is shown in Fig. 3 ▸ for G1, G2 and G3 setups.

Alignment of the optical components is essential to utilize most of the light from the source. The part of the BL1N2 beamline from S1 to S2 is a long monochromatic system with a length of 10000 mm, like many soft X-ray beamlines. During the construction of the beamline, a laser alignment was performed for all optical components from S1 to S2. The mirror mounts of M11, M12, G1, G2 and G3 were arranged on the beamline axis by the laser alignment. Furthermore, the yaw and roll of the grating mirrors were adjusted so that the first- to third-order light diffracted by the grating were aligned in the vertical direction (*Z*-axis) of the beam. Since the optical system containing S1 and S2 was aligned on the beamline axis by the laser alignment, the adjustment of M0 made it easy for monochromatic light to be transported to M2 as the synchrotron radiation beam passes through S1. If M2 does not step off the optical path, monochromatic light is easily transported to the Q point. Due to the careful laser alignment, no pitching, yawing and rolling adjustments have been required, even more than five years after construction.

### 2.3. Energy calibration by photoelectron analyser

A photoelectron analyser is installed in the end-station. It is used not only for photoelectron spectroscopy but also for energy calibration using a continuous calibration function obtained from photoelectron spectroscopy of the inner shell of Au 4*f*_7/2_.

A cleaned surface Au plate is always installed in the sample holder in the measurement chamber. If the kinetic energy of the photoelectron from Au 4*f*_7/2_ measured with a photoelectron analyser is *E*_k_, the binding energy of Au 4*f*_7/2_ is *E*_b_ and the work function of Au is *W*, then the actual incident X-ray energy *E*_a_ is derived as *E*_a_ = *E*_k_ + *E*_b_ + *W*. Literature values are used for *E*_b_ and *W*. Monochromatization using a diffraction grating takes out a certain wavelength depending on the angle of the diffraction grating. Therefore, the grating angle is not a linear function of energy. By obtaining the calibration function of the energy value, it is possible to operate the stepping motor for changing the angle of the gratings by the energy value. The correction of the energy–angle relation is taken into account, including the alignment error of the whole beamline.

### 2.4. XAFS measurements

At the end-station, the total electron yield (TEY) and the partial fluorescence yield (PFY) can be measured. In addition, the photoelectron analyser can measure the Auger electron yield (AEY). Simultaneous measurements of the TEY, PFY and AEY are possible. EXAFS (extended X-ray absorption fine structure) measurements are sometimes performed for Al and Mg.

## 3. Suppression of contamination progress of the mirrors

In the soft X-ray energy region, carbon contamination can easily reduce the beam intensity around the carbon *K*-edge by more than one order of magnitude. At BL1N2, surface contamination was removed by careful ageing by synchrotron radiation at the start of beamline operation in 2015. Ageing by synchrotron radiation refers to ageing by irradiating the optical components installed in the vacuum chamber with synchrotron radiation at lower flux density.

### 3.1. Ageing of the M0 mirror

**Figure 4:**
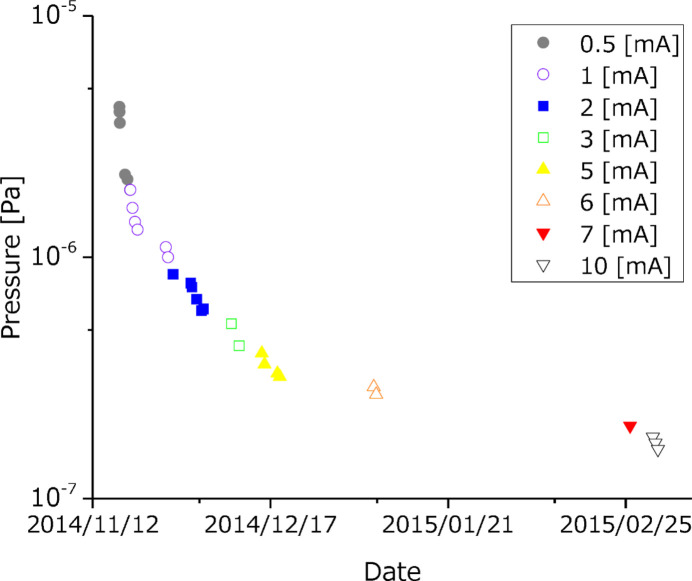
The pressure change during M0 chamber ageing by synchrotron radiation with a ring beam current value of 0.5 mA to 10 mA. The vertical axis shows the pressure on a log scale, and the horizontal axis shows the date. The daily light irradiation time is several hours, and the base pressure with no irradiating light is 1 × 10^−8^ Pa.

BL1N2 has a water-cooled four-dimensional slit upstream of M0. However, since the flux density of synchrotron radiation cannot be lowered by this slit, ageing of M0 of BL1N2 by synchrotron radiation was carried out at a low storage current of 0.5 mA in the test operation phase. The progress of ageing was monitored via the pressure in the vacuum chamber of M0 where the optical components are installed. Fig. 4 ▸ shows the pressure changes after the start of operation. The base pressure without light exposure was 1 × 10^−8^ Pa. When M0 was irradiated by synchrotron radiation for the first time, the pressure increased to 2 × 10^−6^ Pa even for a storage current of 0.5 mA. In the early days of ageing M0, a low storage current from 0.5 mA to 10 mA operation was carried out for several hours a day after the usual user time operation. The ageing of M0 by synchrotron radiation was possible because the beamline construction progressed sequentially from the upstream part, so the time spent waiting for the construction of the downstream part was well utilized.

### 3.2. Ageing of the downstream components

**Figure 5:**
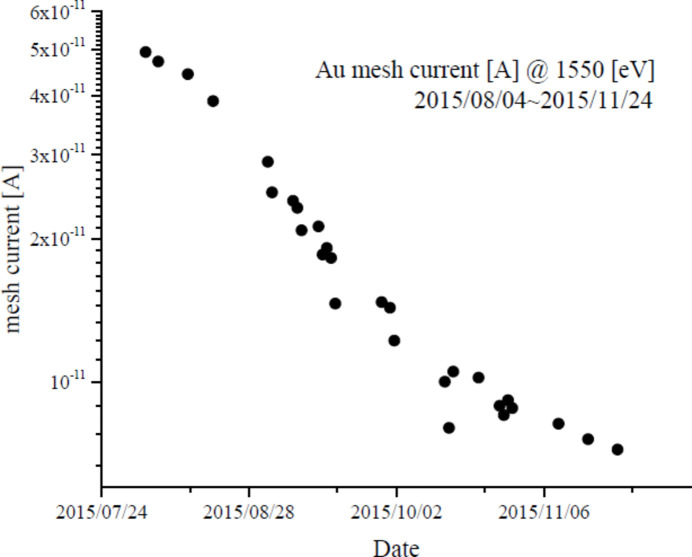
Change in the photocurrent of the Au mesh for *I*_0_ measurement when irradiated with 1550 eV light. It decreased from 5 × 10^−11^ A to 7 × 10^−12^ A over four months.

After the beamline was constructed, the M0 ageing was completed, and M11, G2 and M2 began operation without being aged by synchrotron radiation. M11 and G2 showed a clearly visible dark brown contamination along the synchrotron radiation path. As Fig. 5 ▸ shows, throughput (Au mesh current) of the G2 setup (including M0 and M11) beam intensity decreased by about an order of magnitude over four months of operation. Since M2 was irradiated with light after being monochromated, the amount of light was small and there was no noticeable contamination. The contaminated portion of G2 is avoided, as described later.

**Figure 6:**
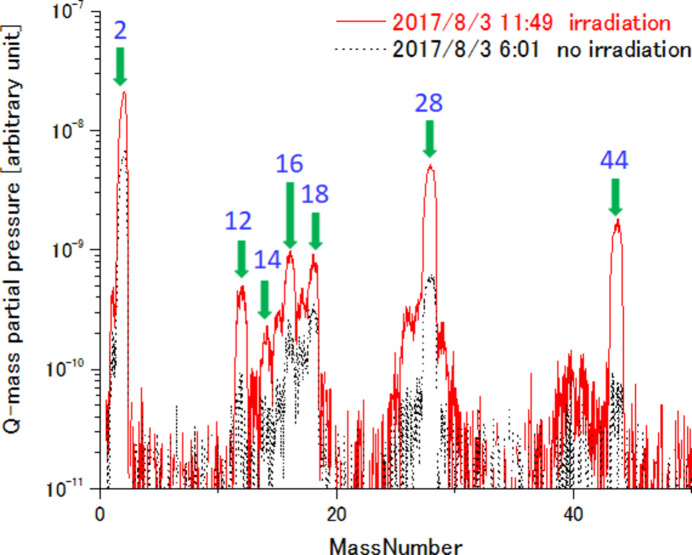
A typical Q-mass spectrum under synchrotron light irradiation on the mirrors.

Since a large flux decrease of G2 was observed at the start of operation in 2015, M12, G1 and G3 were installed after careful ageing by synchrotron radiation. For G1 and G3, controlled synchrotron radiation was irradiated through narrowing the aperture of S1. As mentioned above, the BL1N2 optical system is 1:1 and the spot size of the light on S1 is the same as the light source size. By reducing the aperture of S1, the light density can be changed without changing the footprint on the downstream optical components. This has the same effect as ageing with a low storage current value. In the early stage of ageing, the main beam shutter was opened for a few seconds, and flashing and vacuum recovery were repeated. After that, ageing without flashing was continued. After careful ageing, operation of G1 and G3 started in sequence in 2018. M11, M12 and three grating mirrors are installed in one chamber. Three spatter ion pumps (ULVAC, PST-400AX2) and two NEG pumps (Saes getters, CapaciTorrHV1600) are installed, and the base pressure is 5 × 10^−8^ Pa in the chamber. Fig. 6 ▸ shows a typical Q-mass spectrum under synchrotron light irradiation on the mirrors in the chamber. Under the synchrotron light irradiation, in addition to mass numbers presumed to be CO, CO_2_ and H_2_O, mass numbers presumed to be fragments of organic compounds can be confirmed.

**Figure 7:**
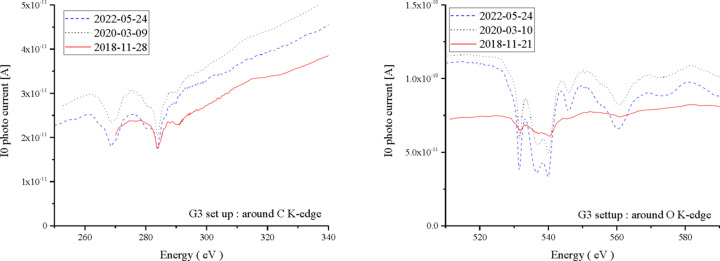
*I*_0_ photocurrent spectra around the *K*-edges of carbon and oxygen. The horizontal axis is the light energy, and the vertical axis is the *I*_0_ current. The solid, dotted and dashed lines are the spectra observed in 2018, 2020 and 2022, respectively. The left and right figures are near the carbon and oxygen *K*-edges, respectively. Note the depth of the dips.

**Figure 8:**
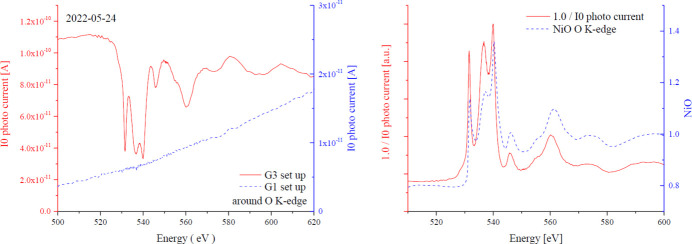
Spectra around the *K*-edge of oxygen. The figure on the left is a comparison of the *I*_0_ photocurrent for the G3 and G1 setups. The right-hand figure is a comparison of the *I*_0_ inverse spectrum in the G3 setup and the oxygen *K*-edge spectrum of NiO.

Fig. 7 ▸ shows a comparison of the *I*_0_ spectra near the *K*-edges of C and O by G3 and M12 in 2018, 2020 and 2022. Regarding C, although there are some changes of flux due to optical adjustment conditions, there is little change in the spectrum by contamination, and the progress of contamination is very slow. As for O, the fine structure gradually appeared after 2018. For the G3 setup, both G3 and M12 are Ni-coated. In the G1 setup both G1 and M11 are Au-coated. As shown in Fig. 8 ▸ (left), the G1 setup shows no oxygen absorption, while the G3 setup shows an absorption (1/*I*_0_) very similar to that of the NiO XAFS spectrum, as seen in Fig. 8 ▸ (right). If the oxygen density of the measurement sample is dilute, the G1 setting may be used because the structure of oxygen in the G3 setup cannot be ignored. However, in the G1 setup, the flux near oxygen is small, so it is rarely used for oxygen measurements. The problem of Ni mirror oxidation remains, but it is a separate issue from contamination. It was shown that, by the ageing at the initial time of use, the attenuation of light due to contamination is suppressed, and furthermore the progress of contamination is suppressed over many years.

### 3.3. Avoidance of G2 mirror contamination

**Figure 9:**
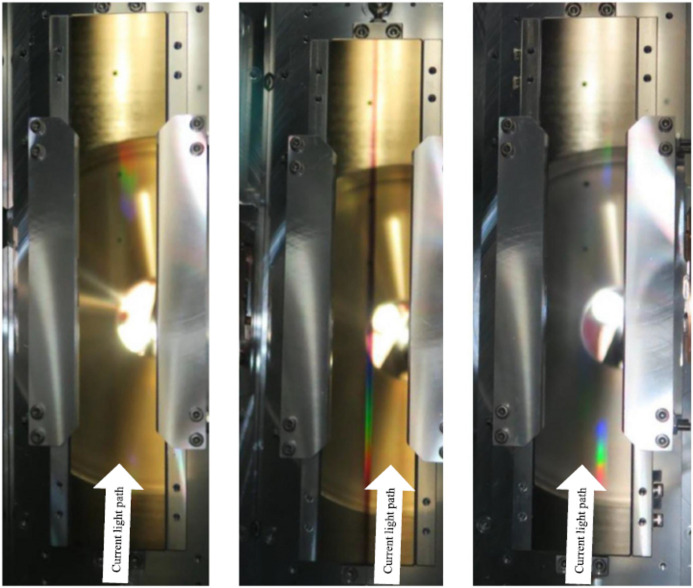
Photographs of, from left to right, G1, G2 and G3. A dark brown linear contaminant can be seen in the centre of G2, where light passed at the early stage of the ageing process. No visible contaminants can be seen on G1 and G3. The outside of the brown line of G2 is used for diffraction. M11 also has a similar brown line as G2.

Since G2 is a planar grating mirror, there are no adverse effects even if it is operated in a position that is horizontally offset from the centre. As for G2, the same ageing as G1 and G3 was carried out for the part that was shifted horizontally to avoid the severely contaminated central area, and it is currently operated with less contamination like G1 and G3. Fig. 9 ▸ shows photographs of G1, G2 and G3 taken in 2022. No visual contaminants are found on the surface of G1 and G3 that were aged carefully by synchrotron radiation. G2 can be seen to have a clear dark brown contamination in the central region.

### 3.4. Contamination effect in the grazing incidence

**Figure 10:**
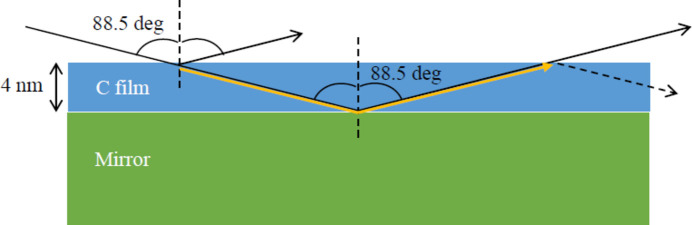
Conceptual diagram of a mirror with a contamination film with lines and arrows as the light path. For simplicity, the lines are drawn assuming that there is no refraction in the contamination film, but the actual light is refracted. The light travels while being absorbed in the C film. The dotted arrows correspond to the lost light that does not reach the Q point.

As shown in Fig. 10 ▸, on the grazing incident mirror, the light path through the contaminant film is largely elongated by a factor 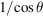 . If the contamination is due to a carbon layer, the refractive index of the carbon layer is so close to 1 that the refraction can be considered negligible. Therefore, the incidence angle of M0 of BL1N2 is 88.5°. The light path length in the film is 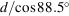 for a film of thickness *d*. At the incident angle of 88.5°, a light decrease of 30% corresponds to a light path of 300 nm, and a deposited contamination film of 4 nm.

## 4. Industrial use

Maintaining stable light quality is important especially for industrial applications. In industry, many comparisons are requested between rejected and accepted products and between improved products and starting items. A comparison is made of measurements recorded several years apart. This comparison is possible only if the measurements are made under the same measurement settings, conditions where the amount of light and the energy of the light are the same as before. For industrial use, stable light quality in the beamline is no longer a requirement but an essential property. Efforts on BL1N2 to optimize industrial use other than light quality are described below.

### 4.1. Dedicated staff support

In order to promote industrial use, AichiSR is trying to assign enough dedicated staff members for each beamline to provide general users with sufficient support during user experiments. At BL1N2, most operations are executed by dedicated staff. This is partly because the vacuum devices of soft X-ray beamlines are complicated and originally designed for individual beamlines. Since the measuring parameters of soft X-ray experiments could vary depending on the sample type and treatment, they have to be determined based on first-look data with enough knowledge of previous experiments. The measurement range and energy step, which determine the measurement time and data quality, need to be chosen carefully to optimize the measurement plan within a given beam time. General users do not need to be XAFS measurement experts of BL1N2 because they can determine the measurement settings using recommendations from dedicated staff who are experienced at XAFS measurements in the energy domain of BL1N2. It is especially helpful for industry users, who tend to use the synchrotron facility as a step in their research and development programs.

### 4.2. Sample holder

**Figure 11:**
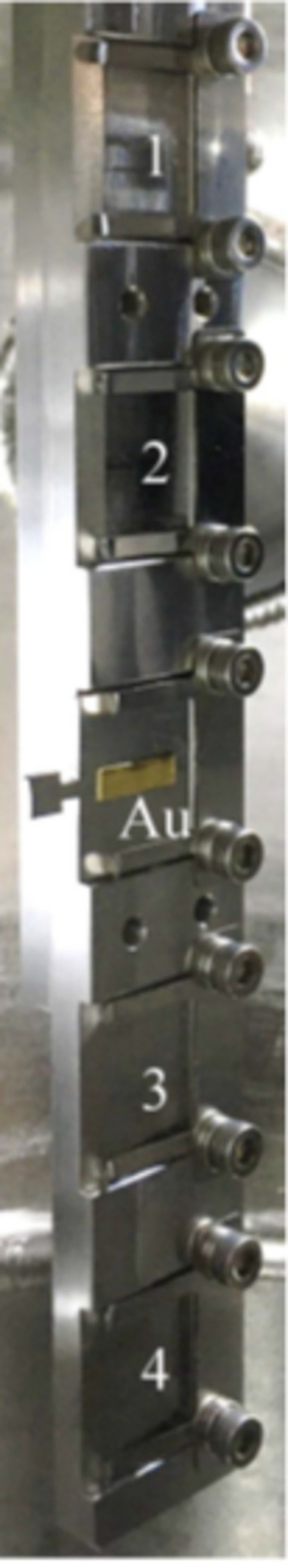
Sample holder. Au is inserted in the middle stage for measurement of the energy correction value. Other stages are empty in this photograph but four sample plates can be inserted into the measurement chamber.

Since the start of operation, the sample holder of the measurement chamber was operated with three sample plates inserted. Then, in response to the need to measure multiple elements with a large number of samples, a holder in which five sample plates can be inserted was adopted in 2019. Fig. 11 ▸ shows a photograph of the sample holder. An Au plate is always inserted in the middle stage for measurement of the energy correction value. The other stages are empty in this photograph. In practice, four samples can be inserted into the holder in the measurement chamber.

### 4.3. Sample bank

For academic use, a few specific samples are measured to obtain very high quality data to lead research in the community. For industrial use, the most frequent measurements are comparisons of data from products under different conditions and methods in the development process. Many similar samples are often measured in a short time. Therefore, the number of samples measured in one use tends to be larger for industrial use. At the beginning of operation in 2015, BL1N2 had only one sample bank in the load lock chamber that could install five sample plates. Since then, another sample bank has been introduced that continues to meet the needs of users and as such seven sample plates could be installed. As of 2022, there are three sample banks, and up to 21 sample plates can be installed in the load lock chamber at one time.

The sample banks can be placed in dedicated vacuum vessels (called ‘transfer vessels’). Samples are directly installed in the load lock chamber in the vessels without exposure to the atmosphere from the user’s lab. The vessels are a standard type used for other vacuum measurement beamlines at AichiSR. Since the sample holders are also of common design, it is possible to measure the same sample in three soft X-ray beamlines at AichiSR without being exposed to the atmosphere. In BL7U, soft X-rays down to 50 eV can be measured, while X-rays up to 6000 eV are available in BL6N1. Therefore, XAFS profiles can be examined at *K*-edges for the elements from Li to V in AichiSR as a one-stop service.

### 4.4. Energy correction measurement

In most cases, there is a difference between the nominal energy and the actual energy. At BL1N2, in all uses, the energy correction value is obtained by photoelectron measurement of the inner shell 4*f*_7/2_ of Au without XAFS measurement of reference samples. In beamlines without the photoelectron analyser, the method for obtaining an energy correction value by XAFS measurement of a reference sample not only requires a long measurement time but a peak fit also needs to be carried out for a specific peak in the reference sample in order to obtain a correction value. It is necessary to obtain the value of the peak from the literature. Moreover, there is no proof that the peak values in the literature are identical to the values recorded at BL1N2. In addition, when a variety of elements need to be measured by this method, a range of reference samples may be required, and sample holder waste occurs, meaning more measurement samples need to be transferred and thus wasted time.

After aligning the grating angle to the starting energy of the XAFS measurement, the Au inner-shell 4*f*_7/2_ peak is measured and the actual energy is obtained (see Section 2.4). For example, for O *K*-edge measurements, if the measured actual energy of light is 500.055 eV for a nominal energy of 500 eV, the correction value is +0.055 eV. For each measurement, the correction value is recorded in the log note together with the measurement file name. After the energy correction value is measured, XAFS measurement is started from this position – there is no worry about motor backlash changing the angle of the grating. Energy correction values are provided to all users for convenience for later analysis.

## 5. Future prospects for industrial use

In XANES (X-ray absorption near-edge structure) experiments, the most popular type of measurement at BL1N2, the observed spectral shape has various characteristics depending on the element. Even for the same element, the spectral features depend on the sample type (for example, whether it is an inorganic or an organic compound). Dedicated staff can recommend the most appropriate measurement parameters to users from first-look rough scan information based on their knowledge of various XANES spectral features they have observed. If various spectral shapes that have been observed in previous experiments were to be stored on computer, machine-learning methods could extract and suggest the best measurement parameters to users. Robotic sample changers may speed up the measurement process, though such automated devices have to be well established in a vacuum. In the future, soft X-ray XAFS measurements could be fully automated, from measurement planning by machine-learning system and automated measurements to basic data measurement.

## 6. Discussion and summary

The outline of the soft X-ray beamline BL1N2 at AichiSR and usage results from 2021 have been described. The optical system arrangement of BL1N2 is characterized by the adoption of grazing-incidence optical systems with an incident angle of 88.4° or more to ensure sufficient light up to 2000 eV. In addition, the optical elements of BL1N2 were carefully aged during the early stage of operation by synchrotron radiation to avoid contaminants on the optics. For the convenience of industrial use at BL1N2, dedicated staff members are assigned, and a sample handling system has been designed to measure multiple samples. With a transfer vessel, users are able to bring samples directly from their labs to the measuring chambers without exposure to the atmosphere. The compatible design of holders and vessels allows samples to be measured at three soft X-ray beamlines in the energy range from 50 to 6000 eV continuously. Future prospects to improve BL1N2 are suggested, such as robotization and auto-optimization of measurement parameters with accumulated knowledge from previous experiments.

## Figures and Tables

**Table:** 

	Shape	Coating	Incident angle (°)	Mirror area (mm)
M0	Toroidal	Au	88.5	500 × 25
M2	Toroidal	Au	88.5	500 × 25

**Table:** 

	Shape	Coating	Included angle (°)	Mirror area (mm)
M11	Spherical	Au	176.8 (for G1, G2)	230 × 20
M12	Spherical	Nickel	174 (for G3)	230 × 20

**Table 2:** Grating mirrors specifications

	Type	Coating	Density (lines mm^−1^)	Mirror area (mm)	Range (eV)
G1	VLS-PGM	Au	500	200 × 20	500–2000
G2	VLS-PGM	Au	1000	200 × 20	1000–2000
G3	VLS-PGM	Nickel	500	200 × 20	150–700

**Table 3:** Focal length of mirrors

	Incidence focal length (mm)		Outgoing focal length (mm)	
	Horizontal	Vertical	Horizontal	Vertical
M0	4000 (from light source)	4000 (from light source)	8500 (center of M0 and M2)	4000 (to S1)
M2	8500 (center of M0 and M2)	3000 (from S2)	3000 (to Q point)	3000 (to Q point)
M11	4926.8 (from S1)		5068.1 (to S2)	
M12	5429.1 (from S1)		4569.5 (to S2)	
